# The disrupted topological properties of structural networks showed recovery in ischemic stroke patients: a longitudinal design study

**DOI:** 10.1186/s12868-021-00652-1

**Published:** 2021-08-02

**Authors:** Yongxin Li, Zeyun Yu, Ping Wu, Jiaxu Chen

**Affiliations:** 1 Formula-Pattern Research Center, School of Traditional Chinese Medicine, Jinan University, Guangzhou, 510632 China; 2 Chengdu University of Traditional Chinese Medicine, Chengdu, 610075 Sichuan China

**Keywords:** Subcortical stroke, Structural connectivity network, Graph theoretical analysis, Topological organization, Longitudinal design

## Abstract

**Introduction:**

Stroke is one of the leading causes of substantial disability worldwide. Previous studies have shown brain functional and structural alterations in adults with stroke. However, few studies have examined the longitudinal reorganization in whole-brain structural networks in stroke.

**Methods:**

Here, we applied graph theoretical analysis to investigate the longitudinal topological organization of white matter networks in 20 ischemic stroke patients with a one-month interval between two timepoints. Two sets of clinical scores, Fugl-Meyer motor assessment (FMA) and neurological deficit scores (NDS), were assessed for all patients on the day the image data were collected.

**Results:**

The stroke patients exhibited significant increases in FMA scores and significant reductions in DNS between the two timepoints. All groups exhibited small-world organization (σ  >  1) in the brain structural network, including a high clustering coefficient (γ  >  1) and a low normalized characteristic path length (λ ≈ 1). However, compared to healthy controls, stroke patients showed significant decrease in nodal characteristics at the first timepoint, primarily in the right supplementary motor area, right middle temporal gyrus, right inferior parietal lobe, right postcentral gyrus and left posterior cingulate gyrus. Longitudinal results demonstrated that altered nodal characteristics were partially restored one month later. Additionally, significant correlations between the nodal characteristics of the right supplementary motor area and the clinical scale scores (FMA and NDS) were observed in stroke patients. Similar behavioral-neuroimaging correlations were found in the right inferior parietal lobe.

**Conclusion:**

Altered topological properties may be an effect of stroke, which can be modulated during recovery. The longitudinal results and the neuroimaging-behavioral relationship may provide information for understanding brain recovery from stroke. Future studies should detect whether observed changes in structural topological properties can predict the recovery of daily cognitive function in stroke.

**Supplementary Information:**

The online version contains supplementary material available at 10.1186/s12868-021-00652-1.

## Background

Stroke is one of the leading causes of substantial disability worldwide. In the world every year, more than 50 million people suffer a stroke [1]. Ischemic stroke generally affect neurological functions, such as motor execution, speech and cognitive function. All these factors negatively impact survivors’ daily activities and quality of life. The healthy life of stroke survivors is lost, and substantial economic costs are needed for poststroke care [2]. One of the challenges facing clinicians in stroke cases is to enhance the level of patient functional recovery. Poststroke recovery is an area in clinical neurology that helps the brain relearn its lost function [3, 4]. Over the past decades, various neuroimaging studies have been applied to understand recovery and plasticity after stroke [5–8]. Brain reorganization plays an important role in the recovery of brain function loss. A growing body of functional neuroimaging studies have shown that cortical changes, such as increased brain activity and changes in functional connectivity, imply the brain with functional reorganization [9–13]. In a previous functional magnetic resonance imaging (MRI) study, stroke patients with good cognitive recovery showed increased brain neural activity only in the default mode network (DMN) and frontotemporal network and decreased brain neural activity in the basal ganglia, which was different from the changes in neural activity of patients with poor cognitive recovery [14]. Structural neuroimaging studies in patients who have had a stroke have also supported the view of cortical reorganization that the structural integrity of the corticospinal tract is reduced in stroke patients [15]. Stroke patients with different degrees of corticospinal tract disruption differ in their motor output. Diffusion MRI measures on white matter (WM) integrity in stroke have found that patients showed a significant decrease of WM integrity in the hemisphere at an early stage. The decreased WM integrity showed further reduction over 3 months compared with healthy controls [7]. Regional variability in stroke patients demonstrated cortical thickness increases particularly in contralesional paracentral, superior frontal and insular regions [16]. Furthermore, dynamic changes in stroke with spontaneous recovery or intervention plasticity have constituted a hot research topic in recent years. From 2 weeks to 3 months poststroke, cortico-cerebellar connectivity and interhemispheric balance in stroke patients showed significant changes [17]. Disrupted brain functional connectivity between paired cortical motor-related regions showed recovery to nearly normal levels in stroke patients under clinical treatment [18]. A longitudinal study of WM integrity in stroke also showed that early dynamic changes of fractional anisotropy (FA) in the corpus callosum within the first 3 months after stroke can predict people’s recovery with the correction of infarct location and extent [7]. Despite these advances in the stroke-related reorganization literatures, little is known concerning how the topological properties of the whole-brain network evolve longitudinally after stroke.

The human brain has been widely conceptualized as a complex system. Previous studies have confirmed that normal neurological function not only depends on the integrity of specific regions but is also contingent on the balanced interplay among multiple regions [19, 20]. The organization of the whole brain network can be examined by a mathematical framework called “graph theory”, which has been used in human brain to investigate the network topological properties. The network topological properties, such as small-world properties, global/local efficiency, and nodal efficiency, are quantified. Graph theoretical analysis in normal human brain have found that the functional and structural brain network exhibits a “small-world” organization [20]. Previous studies have shown that the human brain network has a highly efficient small-world topology: high local clustering of nodes and minimal path lengths between the nodes [19]. In the growing field of network science, changes in functional and structural brain networks have been applied in a variety of conditions, such as stroke [21, 22] and epilepsy [23]. Analysis of functional and structural networks in stroke cases has proven that graph theory is a particularly useful tool for assessing changes in brain topological patterns [22, 24–27]. Stroke patients showed a breakdown in network topological properties including the sensorimotor network and frontoparietal control system, which correlated with the severity of motor deficits [27]. Dynamic changes in the topological configuration of the functional network demonstrated consistent alterations over time [25]. Significant changes in regional centralities were observed within the motor execution network. Currently, most previous graph theory analyses of stroke have focused on the functional network properties, which can further enable us to understand functional reorganization following stroke. Relatively few neuroimaging studies have applied graph theoretical analysis to examine longitudinal changes in structural network properties following stroke. Previous study of stroke have shown that the coupling of functional and structural connectivity networks decreased in the stroke state and that the degree of coupling was related to the Fugl-Meyer motor assessment (FMA) scores of stroke patients [22]. Thus, examining the longitudinal changes in structural network topology properties would complement previous observations of brain recovery after stroke and provide insight into the understanding of the organization principles of human brain structural networks.

The objective of the present study was to apply graph theory to diffusion tensor images of ischemic stroke patients to (1) construct structural connectivity network for the brain, (2) analyze the longitudinal track of its underlying topological properties and (3) further explore correlations between the values of topological properties in regions with significant changes and clinical variables. To do this, a group of ischemic stroke patients was recruited. Longitudinal changes in the topological organization of whole-brain structural networks in stroke patients were constructed, and these networks were compared to those in healthy subjects. Given the previous evidence of previous neuroimaging studies in stroke, we hypothesized that adults with ischemic stroke would show disruption of topological properties in the regions related to sensorimotor function and the DMN. We further hypothesized that these topological changes in the patient group would be partially restored with time and significantly correlated with clinical variables.

## Methods

### Subjects

The present study included twenty first-ever stroke patients (all right-handed, mean age: 65.25  ±  12.31 years) with unimanual motor deficits due to subcortical ischemic lesions who were recruited from the First Affiliated Hospital of Chengdu University of Traditional Chinese Medicine, China. The inclusion criteria for patients were as follows: (1) first-ever ischemic stroke; (2) strictly subcortical lesions; (3) absence of neglect, aphasia, or dementia; (4) no other neurological or psychiatric disorders; and (5) within 5 months after the onset. Before enrolling in this study, no patients underwent other experimental therapies. After enrollment in this study, all patients were diagnosed and treated with enteric-coated aspirin (100 mg daily). All patients were also intravenously injected with citicoline (0.5 g daily). The main demographic and clinical information of all participants were summarizes in Table 1. Diffusion tensor imaging (DTI) was performed twice on all patients with a one-month interval: once before and one month after enrollment in this study. FMA and neurological deficit scores (NDS) were assessed for all patients on the day the MRI data were collected. The FMA can be used to evaluate motor function after stroke, and the NDS can be used to test the severity of neurological functional deficits in stroke patients. Paired t tests were used to detect whether these clinical scores changed between the two timepoints. Fourteen healthy controls (HC, all right-handed, mean age: 61.00  ±  10.84 years) without psychiatric illnesses or neurological disorders were recruited. All control subjects were scanned only once on the day when they were recruited.

**Table 1:** Patient characteristics

Patient no.	Age (yr)	Gender	Affected hand	Lesionl	Lesion size (cm^3^)	Lesion duration (days)	FMA_1st	FMA_2nd	NDS_1st	NDS_2nd
1	69	F	R	L Pons/CS	0.35	135	90	96	13	5
2	75	F	R	L TH	0.41	22	74	90	32	17
3	73	M	R	L BG/Bi CS	1.13	132	80	84	26	23
4	43	F	R	L CN/Bi PV	0.45	25	92	96	15	6
5	71	F	L	R BG	2.24	32	88	95	21	10
6	62	M	R	L BG/CS	0.20	36	82	95	25	11
7	73	M	R	L BG/CS	0.54	26	84	91	24	18
8	81	M	R	L BG/IC	1.35	22	83	92	23	12
9	49	M	R	L BG/CS	0.38	56	85	93	24	16
10	67	M	R	L BG/R PV	1.18	33	80	93	26	12
11	78	M	R	L BG/PHLV	3.12	21	84	95	19	9
12	70	F	R	L CS/PV	0.25	23	85	90	22	14
13	74	F	R	L PV	0.12	148	90	96	20	12
14	37	M	R	L TH/LN	1.89	148	88	94	24	14
15	79	M	L	R BG	0.29	50	85	92	26	15
16	60	F	R	L BG/Bi RA	1.26	23	80	85	28	20
17	73	M	R	L CE/PV	2.09	56	77	90	31	20
18	54	F	R	L BG	1.19	45	80	85	26	23
19	54	F	R	L BG	0.28	23	86	92	24	12
20	63	M	R	L TH	0.29	21	82	85	25	18
Summary	65.25 ± 12.31	9F/11 M	2L/18R			53.85 ± 46.12	83.75 ± 4.56	91.45 ± 3.97	23.70 ± 4.60	14.35 ± 5.07
Control	61.00 ± 10.84	6F/8 M								

Summary values are reported as the means  ±  standard deviation. Lesion duration refer to how long the patients were enrolled after the stroke

*BG* basal ganglia; *Bi* bilateral; *CE* capsula externa; *CN* caudate nucleus; *CS* centrum semiovale; *DTI*, diffusion tensor imaging; *F* female; *FMA* Fugl-Meyer motor assessment; *IC* internal capsule; *L* left; *LN* lenticular nucleus; *M* male; *NDS* neurological deficit scores; *PHLV* posterior horn of lateral ventricle; *PV* periventricular; *R* right; *RA* radial area; *TH* thalamus

After a detailed explanation of the study purpose, procedure, possible risks and discomforts, all participants agreed to participate in the study and written informed consent was obtained for each subject. This study was approved by the Ethics Committee of Chengdu University of Traditional Chinese Medicine (No. 2011KL-002) and was conducted in accordance with the Helsinki Declaration.

### Data acquisition

The MRI data were acquired using a 3.0-Tesla Siemens scanner (MAGNETOM Trio Tim, Siemens, Germany) at the West China Hospital MRI Center, Chengdu, China. Foam cushions were used to minimize head movement of each participant during the scanning. First, a T1-weighted 3D structural MRI was obtained for each subject with the following parameters: repetition time  =  1900 ms, echo time  =  2.26 ms, flip angle  =  9°, slices  =  176, field of view  =  256 mm  ×  256 mm, and voxel size  =  1  ×  1  ×  1 mm^3^. Then, diffusion-tensor images were acquired using a spin-echo planar imaging sequence with the following parameters: repetition time  =  6800 ms, echo time  =  93 ms, field of view  =  240  ×  240 mm^2^, in plane resolution  =  1.875  ×  1.875 mm^2^, slice thickness  =  3 mm, 50 axial slices, and 30 diffusion-weighted images (b  =  1000 s/mm^2^). The diffusion-weighted images were acquired in blocks of 2 images with no diffusion weighting (b_0_). All participants were instructed to remain awake, and lie motionless without thinking about anything specific during the entire scan.

### Data preprocessing

Before data preprocessing, images from two patients with right-sided stroke (No. 5 and No. 15) were transposed across the midsagittal plane, thereby lateralizing the lesions to the left hemisphere in all patients [28]. The DTI data of two control subjects matched to the two patients were selected and transposed across the midsagittal plane to reduce the error caused by this transformation. After this process, for all patients, the left side corresponded to the ipsilesional hemisphere. Then, DTI data analysis was performed by tools from FSL (www.fmrib.ox.ac.uk). The preprocessing procedures were as follow: first, images were corrected for eddy-current distortions and head motion using affine registration [29] and nonbrain tissue was removed using BET [30]. Then, FA for each voxel was calculated by using the DTIFIT tool from the FSL package. The preprocessing data were inputted into the DCP pipeline toolbox to examine the diffusion connectome [31]. Deterministic fiber tracking was performed by applying the “dti_tracker” command in the diffusion toolkit. Tracking was halted when FA was below 0.1 or turning angle  <  45°.

### Network definition and topological analysis

We employed the automated anatomical labeling (AAL) template to define the nodes [32]. This atlas requires transformation to the native DTI space from the standard space of each individual. To achieve this purpose, the following steps were performed. First, the individual 3D T1-weighted images were co-registered to the corresponding individual b0 image in DTI space. Then, the transformed 3D T1-weighted images were nonlinearly transformed to the ICBM152 T1 template in Montreal Neurological Institute (MNI) space. After this step, inverse transformations were applied to warp the AAL atlas from the MNI space to the DTI native space. To do this, we obtained 90 cortical and subcortical regions, each region representing a node of the structural network. Each pair of brain regions as define above was considered structurally connected if there was at least one fiber streamline with one endpoint located in each of these two regions. Based on the linking fibers, an FA-weighted matrix was calculated for each subject. Each row or column in a matrix represents a brain region. The values of the elements in this matrix represent FA averaged between paired regions. Thereafter, a FA-weighted 90  ×  90 structural network was produced for each subject.

For the structural matrices, a threshold of connection sparsity, S, was defined as the ratio of the number of actual edges divided by the maximum possible number of edges in the network. All resulting networks have the same number of edges by using this approach. Currently, there is no criterion for selecting a single threshold for constructing structural brain networks. In this study, a range of sparsity thresholds from 0.1 to 0.5 with an interval of 0.01 was applied to allow the structural network properties to be properly estimated. The networks should not fragment in either group at the lower bound of the range. For densities above 0.45, the networks became increasingly random (σ  <  1.5) [33]. The reference showed that the small-world model synchronized as rapidly as a fully random network for thresholds  >  0.5. Therefore, the maximum threshold was selected at 0.5 in this study to ensure the thresholded network displayed small-worldness. Two regions were considered connected if the connection sparsity was more than a given threshold. According to this process, the individual structural connectivity matrix was converted into a binary matrix. Graph theory were then performed on the binary matrices to calculate the topologic properties of brain structural connectomes for each subject at both global and nodal levels across the sparsity range using GRETNA software (http://www.nitrc.org/projects/gretna/) [34].

Network analyses were performed at different levels, including analyses of global network metrics, regional nodal properties, and local connections. The global metrics included: clustering coefficient (C_p_), normalized clustering coefficient (γ), characteristic path length (L_p_), normalized characteristic path length (λ), and small-worldness (σ) and network efficiency [global efficiency (E_g_) and local efficiency (E_loc_)]. The regional nodal graph metrics were assessed as the involvement of nodal efficiency and nodal local efficiency. For a network (G) with N nodes, the C_p_ is calculated as

$$C_{p} \left( i \right) = \frac{1}{N}\mathop \sum \limits_{i \in G} \frac{{E_{i} }}{{k_{i} \left( {k_{i} - 1} \right)/2}}$$

where k_i_ is the number of edges by node i, and E_i_ is the number of edges existing in G_i_, the subgraph consisting of the neighbors of node i. The Cp of a network indicates the extent of local interconnectivity in a network.

The L_p_ is calculated as follows:

$$L_{p} \left( G \right) = \frac{1}{{\frac{1}{{N\left( {N - 1} \right)}}(\mathop \sum \nolimits_{i \ne j \in G} \frac{1}{{L_{ij} }})}}$$

where L_ij_ is the shortest path length between the pair of nodes i and j. The mean of the shortest path between each pair of nodes were defined as characteristic shortest path length Lp, which can be used to quantifies the ability for information to be propagated in parallel.

The normalized clustering coefficient $$\gamma = {\raise0.7ex\hbox{${C_{p}^{real} }$} \!\mathord{\left/ {\vphantom {{C_{p}^{real} } {C_{p}^{rand} }}}\right.\kern-\nulldelimiterspace} \!\lower0.7ex\hbox{${C_{p}^{rand} }$}}$$ and the normalized characteristic path length $${\uplambda } = {\raise0.7ex\hbox{${L_{p}^{real} }$} \!\mathord{\left/ {\vphantom {{L_{p}^{real} } {L_{p}^{rand} }}}\right.\kern-\nulldelimiterspace} \!\lower0.7ex\hbox{${L_{p}^{rand} }$}}$$ were also computed to examine the small-worldness properties of the structural network. In this formula, the $$C_{p}^{real}$$ and $$L_{p}^{real}$$ are the clustering coefficient and the characteristic path length of real networks, respectively. The $$C_{p}^{rand}$$ and $$L_{p}^{rand}$$ represent the means Cp and the mean Lp derived from 100 matched random networks. A real network would be considered small-world if γ  >  1 and λ $$\approx 1$$ or $$\sigma = \frac{\gamma }{{\uplambda }} > 1$$. Thus, a small-world network has not only the higher local interconnectivitybut also theapproximately equivalent shortest path length compared with the random networks.

The The global efficiency E_g_ of network G is calculated as:

$$E_{g} \left( G \right) = \frac{1}{{N\left( {N - 1} \right)}}\mathop \sum \limits_{i \ne j \in G} \frac{1}{{L_{ij} }}$$

where L_ij_ is the shortest path length between each pair of nodes i and j.

The local efficiency E_loc_ of network G is computed as:

$$E_{loc} \left( G \right) = \frac{1}{N}\mathop \sum \limits_{i \in G} E_{glob} \left( {G_{i} } \right)$$

where G_i_ denotes the subgraph composed of the nearest neighbors of node i. This indexreveals how much the network is fault tolerant.

We also computed the nodal efficiency and nodal local efficiency to determine the nodal characteristics of the networks.

The nodal efficiency is defined as:

$$nE\left( i \right) = \frac{1}{N - 1}\mathop \sum \limits_{i \ne j \in G} \frac{1}{{L_{ij} }}$$

where L_ij_ is the shortest path length between each pair of nodes i and j. This index measures the average shortest path length between a given node i and all the other nodes in this structural network.

The nodal local efficiency is defined as:

$$nE_{loc} \left( {\text{i}} \right) = E_{g} \left( {G_{i} } \right)$$

where G_i_ denotes the subgraph composed of the nearest neighbors of node i.

To provide a summarized scalar independent of single threshold selection [35], we also calculated the area under the curve (AUC) for each network metric (global and local topological properties). The AUC for a general metric σ was calculated in this study over the sparsity range from 0.1 to 0.5 with an interval of ${ }0.01$.

### Statistical analysis

The longitudinal changes of clinical cognitive scores including FMA and NDS between the two timepoints in the patients were analyzed using paired t tests. For the AUCs of the global network metrics, one-way ANOVA was used to compare the differences among the groups with age and sex as covariates (p  <  0.05). For significant ANOVA results, post-hoc between-group differences in the AUCs of global network graph metrics were analyzed (p  <  0.05). Group comparisons of nodal network metrics were performed by one-way ANOVA with age and sex as covariates, and post-hoc pairwise comparisons were performed if ANOVA yielded significant results (p  <  0.05). BrainNet Viewer software was used to visualize the significant results of nodal metrics [36].

We then calculated correlations between the structural network metrics and the clinical variables (FMA and NDS) in the patient group (p  <  0.05), removing the effects of age and sex. Here, we mainly focused on topological metrics, such as Cp, nodal efficiency, and nodal local efficiency. For these network metrics, we only selected the nodes that met the following criteria to identify areas of interest. First, the topological metrics for a node showed a significant change in the patients at the first timepoint compared with the HCs. Second, the longitudinal changes in topological metrics at this node were significant between the two timepoints in the patients. Third, the topological metrics at this node at the second timepoint were no longer significantly different from metrics for theHCs. The correlation results were corrected by the Bonferroni method, based on the number of areas that we selected.

## Results

### Demographic and clinical characteristics

No significant differences in age (p  =  0.306) or sex (p  =  0.901) were observed between the stroke patients and the HCs. For clinical variables, the FMA scores increased significantly (pairwise t  =  − 9.489; p  <  0.001) from 83.75  ±  4.56 (the first timepoint) to 91.45  ±  3.97 (the second timepoint). The stroke patients exhibited significant improvement in motor function, as measured by FMA, from the first to the second timepoint. The NDS scores declined significantly (pairwise t  =  12.870; p  <  0.001) from 23.70  ±  4.60 (the first timepoint) to 14.35  ±  5.07 (the second timepoint). Stroke patients also showed significant recovery of the neurological function as measured by the NDS from the first to the second timepoint. In Additional file 1: Figure S1, the changes in clinical variables are shown in detail. The duration from stroke onset to the first MRI scan ranged from 21 to 148 days (mean value, 53.85  ±  46.12 days).

### Groupwise differences in AUC values of global network metrics

**Fig. 1:**
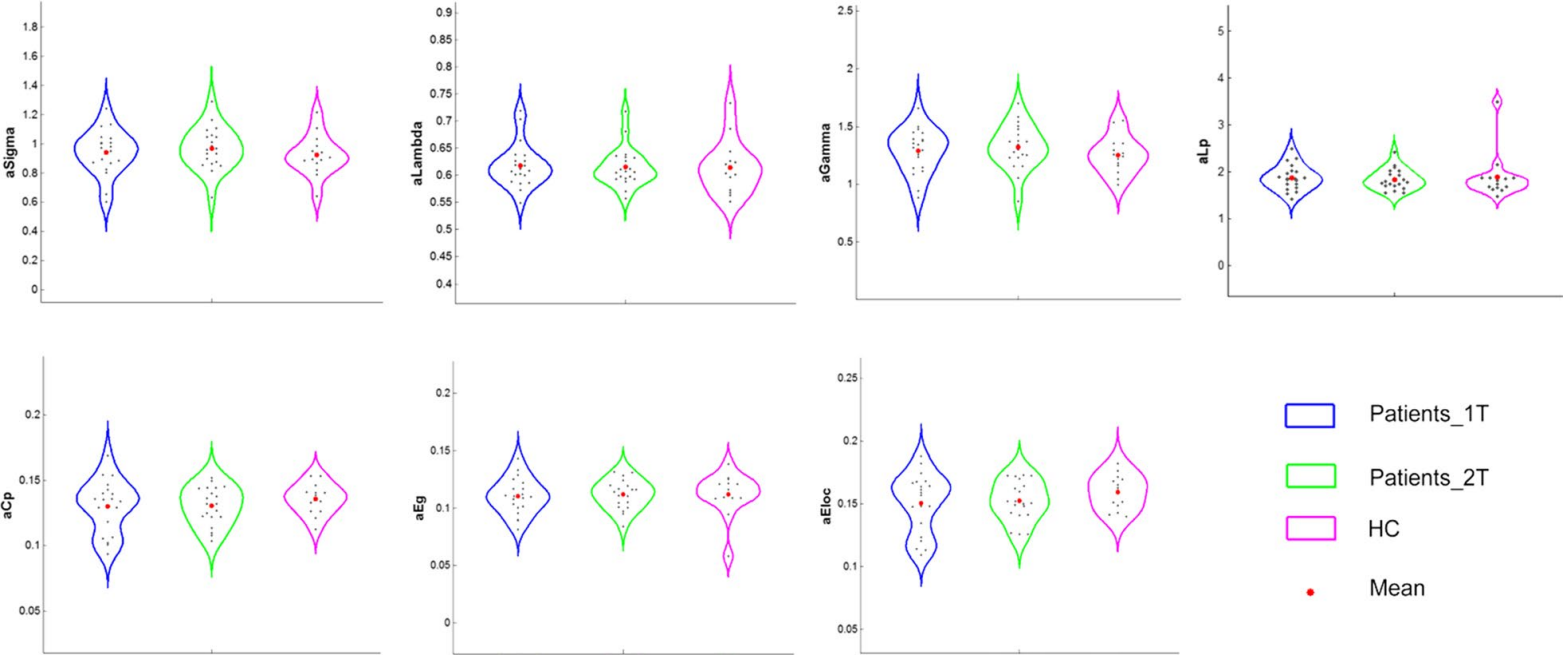
Group comparisons of AUC values of global nework properties between groups. *aE*_*g*_ AUC values of global efficiency; *aE*_*loc*_ AUC values of local efficiency; *aC*_*p*_, AUC values of clustering coefficient; *aLp* AUC values of characteristic path length; *aSigma* AUC values of normalized characteristic path length; *aLambda* AUC values of normalized clustering coefficient; *aGamma* AUC values of small-worldness; *Patients_1T* patient group at the first timepoint; *Patients_2T* patient group at the second timepoint

Both stroke patients and HCs showed a small-world organization of brain white matter networks characterized by high C_p_ (γ  >  1) and similar Lp (λ ≈ 1). Moreover, stroke patients also maintained the small-world topology of their brain structural connectome between the two timepoints. No significant differences in any global network metrics were detected between the HCs and the two patient groups (see Fig. 1).

### Alterations in the regional properties of structural network in strokes

**Fig. 2:**
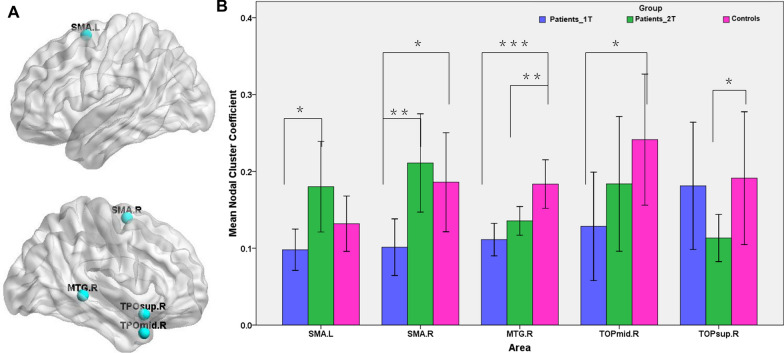
Brain regions showing significant changes of Cp in brain structural networks among groups. **A** The location of nodes with significant changes in Cp; **B** the differences in Cp between patients and HCs and between the two time-point in patients. *p  <  0.05; **p  <  0.01, ***p  <  0.001. *SMA* supplementary motor area; *MTG* middle temporal gyrus; *TOPmid* temporal pole of the MTG; *TOPsup* temporal pole of the superior temporal gyrus

Further statistical analysis revealed that regional topological organization between groups showed significant differences (p  <  0.05). Compared with the HCs, patients had significant reduced Cp mainly in the right supplementary motor area (SMA), right middle temporal gyrus (MTG) and right temporal pole of the MTG (Fig. 2A). Approximately one month later, Cp values in the right SMA and right temporal pole of the MTG no longer showed significant differences between the stroke patients and the HCs. Paired t tests found that Cp in the bilateral SMA increased significantly in the stroke patients from the first to the second timepoint (Fig. 2B). All regions showing significant differences in Cp between groups are shown in Fig. 2A, and detailed differences are listed in Fig. 2B.

**Fig. 3:**
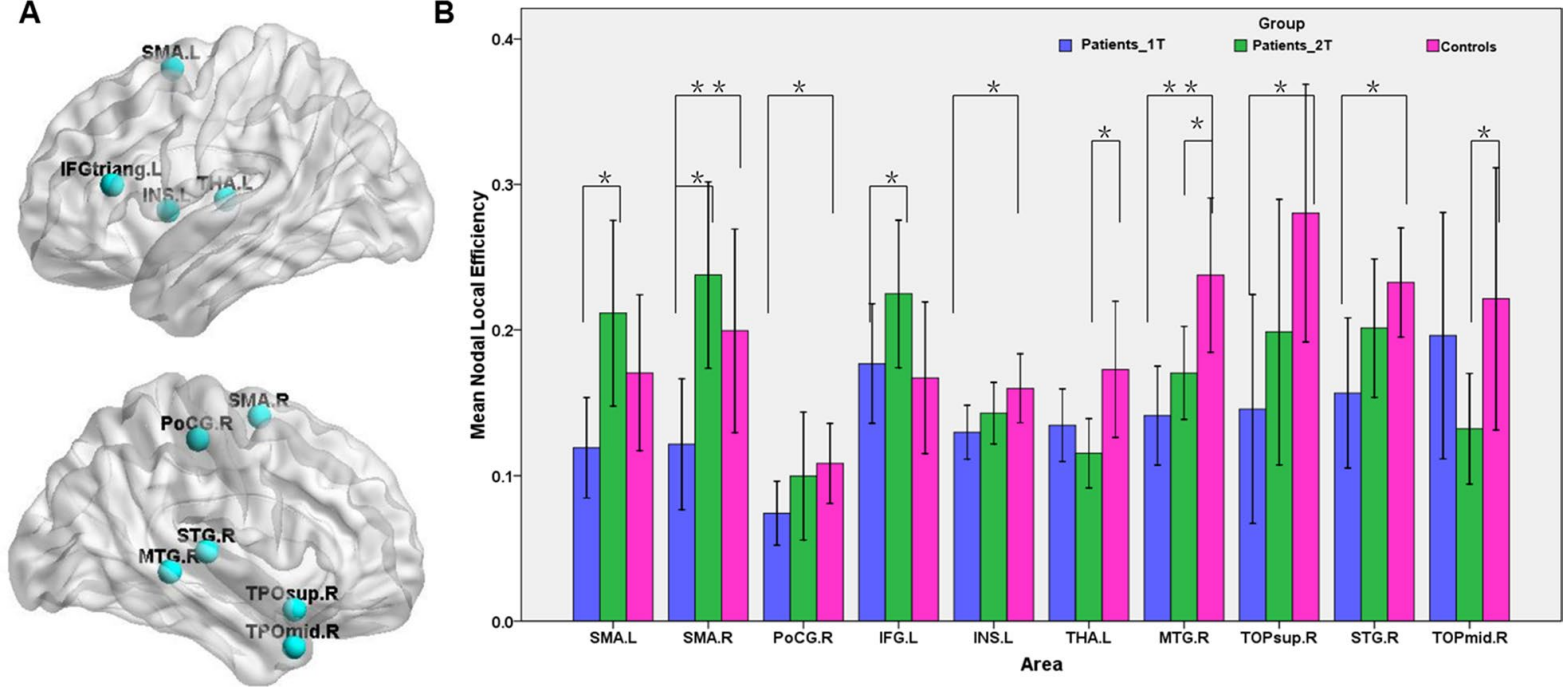
Brain regions showing significant changes of nodal local efficiency in brain structural networks among groups. **A** The location of nodes with significant changes in nodal local efficiency; **B** the differences in nodal local efficiency between patients and HCs and between the two time-point in patients. *p  <  0.05; **p  <  0.01. *SMA* supplementary motor area; *PoCG* postcentral gyrus; *IFG* inferior frontal gyrus; *INS* insular; *THA* thalamus; *MTG* middle temporal gyrus; *TOPmid* temporal pole of the MTG; *STG* superior temporal gyrus; *TOPsup* temporal pole of the STG

Compared with the HCs, the stroke patients had significant reduced nodal local efficiency mainly in the right SMA, right postcentral gyrus, left insula, right superior temporal gyrus (STG), right MTG and right temporal pole of the right STG (Fig. 3A). Approximately one month later, the nodal local efficiency in all the above regions except the right MTG no longer differed significantly between the stroke patients and the HCs. Paired t-tests also showed that the nodal local efficiency increased significantly in the bilateral SMA and left inferior frontal gyrus (IFG) of the stroke patients from the first to the second timepoint (Fig. 3B).

**Fig. 4:**
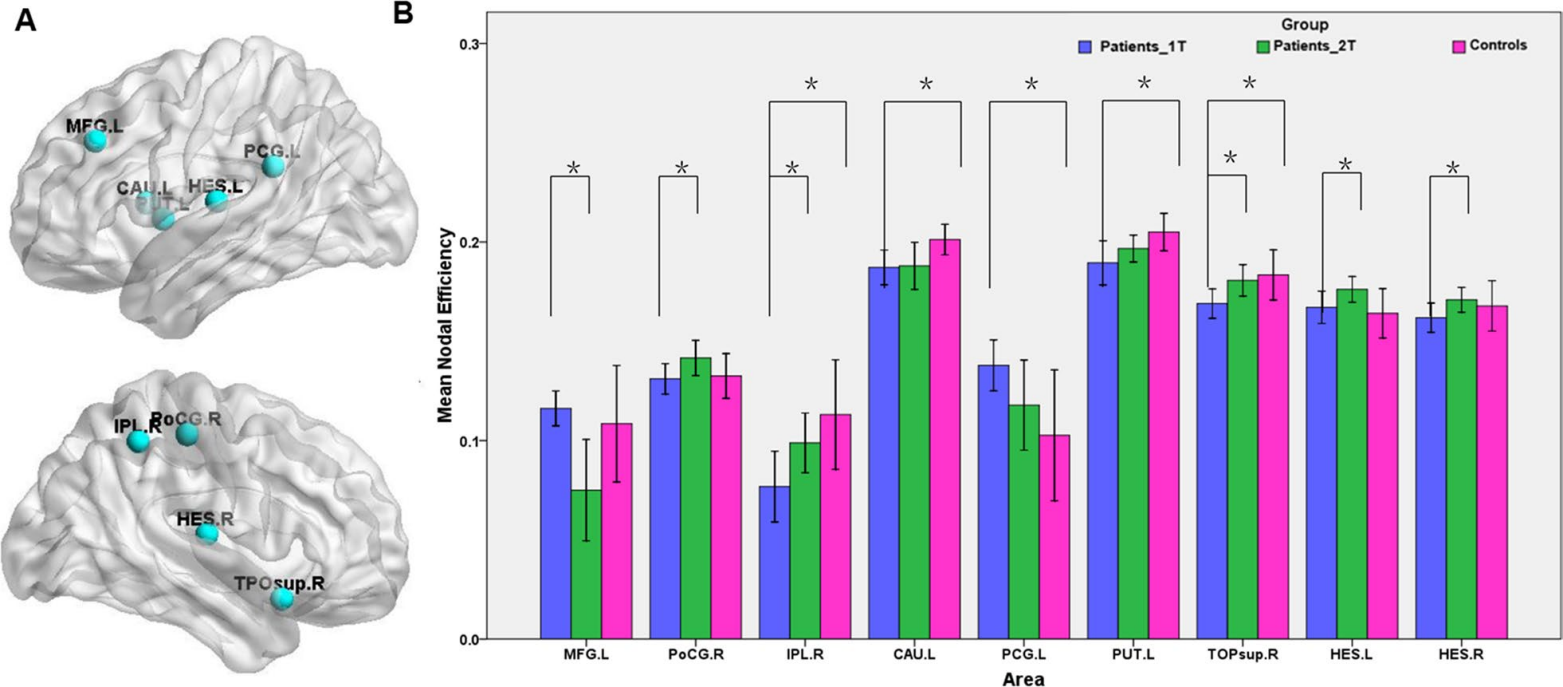
Brain regions showing significant changes of nodal efficiency in brain structural networks among groups. **A** The location of nodes with significant changes in nodal efficiency; **B** the differences in nodal efficiency between patients and HCs and between the two time-point in patients. *p  <  0.05. *MFG* middle frontal gyrus; *PoCG* postcentral gyrus; *IPL* inferior parietal lobe; *CAU* caudate; *PCG* posterior cingulate gyrus; *PUT* putamen; *TOPsup* temporal pole of the STG; *HES* Heschl’s gyrus

Decreased nodal efficiency were found in the left caudate, left putamen, right inferior parietal lobe (IPL) and temporal pole of the STG in the stroke patients (Fig. 4A). Significant increases in nodal efficiency were found in the left posterior cingulate gyrus (PCG) in stroke patients. Approximately one month following stroke, the nodal efficiency in all these regions no longer differed significantly between the patients and the HCs. Paired t tests demonstrated that the nodal efficiency in the right postcentral gyrus, right IPL and bilateral Heschl’s gyrus in the stroke patients increased significantly from the first to the second timepoint (Fig. 4B). The nodal efficiency in the left middle frontal gyrus showed a significant decrease from the first to the second timepoint.

### Relationships between structural network metrics and clinical variables

**Fig. 5:**
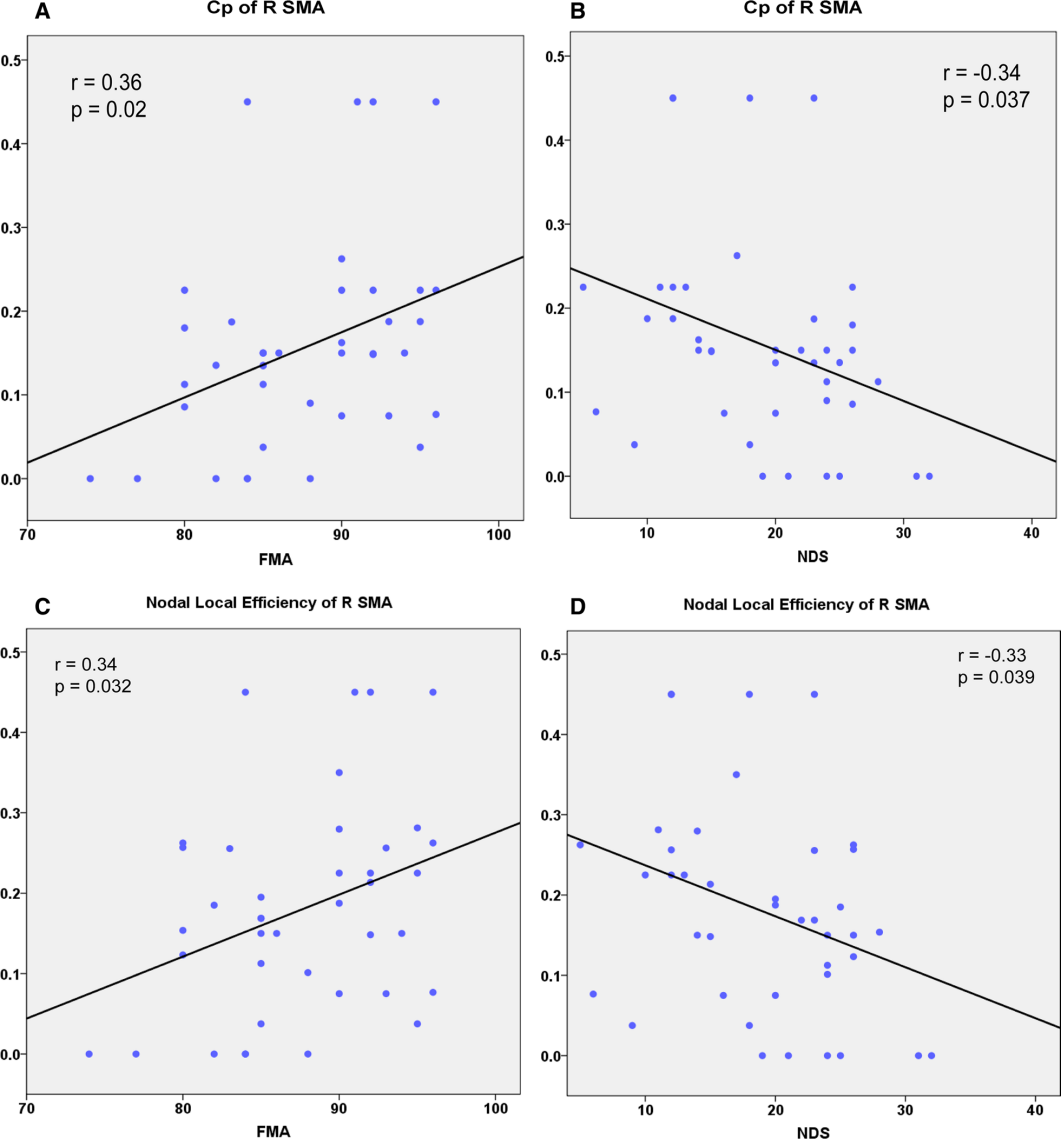
**A** Scatterplot showing significant positive correlation between Cp of the right SMA and FMA in stroke patients; **B** scatterplot showing significant negative correlation between Cp of the right SMA and NDS in stroke patients; **C** scatterplot showing significant positive correlation between nodal local efficiency of the right SMA and FMA in stroke patients; **D** scatterplot showing significant negative correlation between nodal local efficiency of the right SMA and NDS in stroke patients. *SMA* supplementary motor area; *FMA* Fugl-Meyer motor assessment; *NDS* neurological deficit scores

**Fig. 6:**
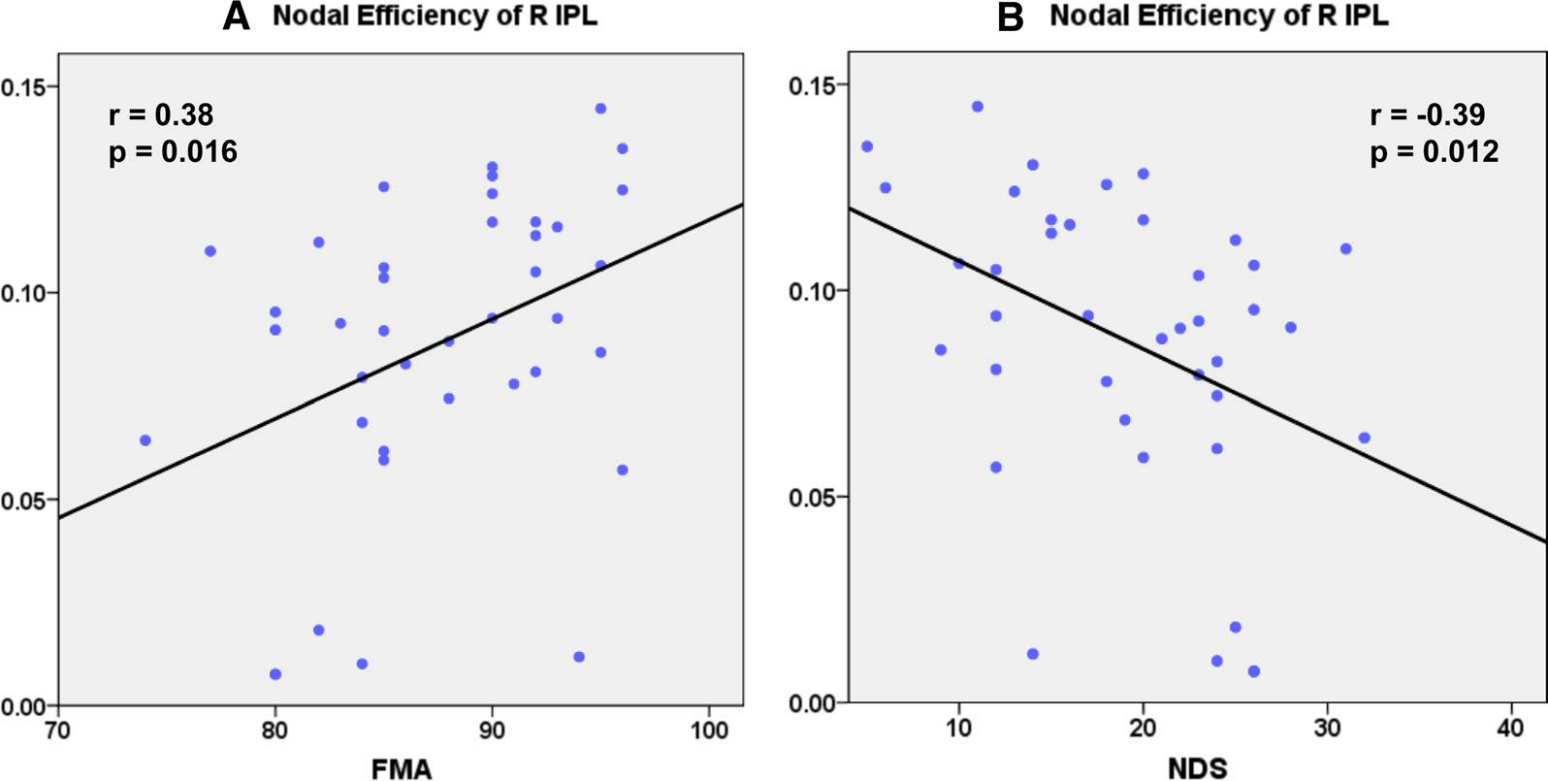
**A** Scatterplot showing significant positive correlation between nodal efficiency of the right IPL and FMA in stroke patients; **B** scatterplot showing significant negative correlation between nodal efficiency of the right IPL and NDS in stroke patients. *IPL* inferior parietal lobe; *FMA* Fugl-Meyer motor assessment; *NDS* neurological deficit scores

From the statistical results for Cp and nodal local efficiency, only the right SMA met the criteria for our areas of interest: the topological metrics (Cp and nodal local efficiency) in the right SMA showed a significant increase from the first to the second timepoint and were not significantly different from metrics for the HCs. We found significant correlations between the Cp of the right SMA and the clinical variables (FMA: r  =  0.36, p  =  0.021; NDS: r  =  − 0.34, p  =  0.037, Fig. 5A, B; p <  0.05). We also found significant correlations between the nodal local efficiency of the right SMA and the clinical variables (FMA: r  =  0.34, p  =  0.032; NDS: r  =  − 0.33, p  =  0.039, Fig. 5C, D; p <  0.05). Additionally, for the statistical results for nodal efficiency, both right IPL and right temporal pole of STG meet the criteria as our interesting areas. The nodal efficiency of right IPL was correlated with the clinical variables (FMA: r  =  0.38, p  =  0.016; NDS: r  =  − 0.39, p  =  0.012, Fig. 6A, B; p <  0.025, Bonferroni correction 0.05/2  =  0.025). The nodal efficiency of the right temporal pole of the STG showed no significant correlations with the clinical variables.

## Discussion

In the present study, graph-based theoretical approach was used to investigate the topological characteristics of the brain structural connectomes in adults with ischemic stroke. Stroke patients and HCs showed small-world properties of the structural networks characterized by γ  >  1 and λ ≈ 1. Despite the common small-world topology, stroke patients also exhibited significantly lower values of Cp and nodal local efficiency in the right SMA, right MTG and temporal pole of the MTG. Longitudinal research showed that these decreased network metrics recovered after a month and showed no significant differences compared with the HCs. Furthermore, the nodal efficiency of stroke patients was reduced in the left caudate, left putamen and right temporal pole of the STG and elevated in the left PCG. Longitudinal comparison in stroke patients also found that nodal efficiency increased in the right postcentral gyrus and right IPL after a month. The Cp and nodal local efficiency in the right SMA were positively correlated with the FMA scores and negatively correlated with the NDS scores. Node efficiency in the right IPL also showed a significant correlation with clinical variables. Together, our study showed that the brain network architecture of adults with ischemic stroke was changed at nodal levels. Our results provide preliminary evidence that the disrupted topological organization of the structural network in stroke patients may improve during the recovery process.

### Distributed regional topological organization after stroke

The human brain is a complex system that is capable of generating and integrating information from multiple sources with high efficiency [37]. Characterization of the topological architecture of the structural networks is crucial in understanding the neural mechanism in brain disorder. Graph theory method can provide a framework to characterize network topology. Previous WM network studies in healthy adults have found that the resultant WM network exhibits small-worldness [38, 39]. Previous network studies in large-scale brain functional and structural connectivity have also revealed a small-world property of the brain network in healthy human [19, 20, 40]. Previous studies have also found that patients with ischemic stroke demonstrated small-world functional and structural networks [22, 26, 27, 41]. In the present study, stroke patients also showed small-world properties of their structural networks at both timepoints. Consistent with the graph theory results of these previous neuroimaging studies, structural connectivity networks of the stroke patients and HCs showed small-world organization in the present study. The graph theory results indicated that normal human brain network has high efficiency in information processing and transfer [42]. This suggests that the human brain integrate the information across segregated brain regions. The brain is organized with an optimal balance of integration and segregation, even in the disease state after stroke.

Despite the common small-world topology of the structural networksin all groups, the stroke patients showed significant changes in Cp and nodal local efficiency. Cp is a network parameter which indicates the extent of local interconnectivity or cliquishness in a network. Local efficiency is a network parameter reveals how much the network is fault tolerant. One main finding of the present study is that stroke patients showed significant decrease in both Cp and nodal local efficiency in the right SMA and right MTG. The simultaneous decrease in both network indexes indicates that the network topological organization of information transfer across these regions was altered in stroke patients. Decreased nodal topology in the SMA for patients with stroke could be interpreted in the context of decreased cortico-cortical connectivity within the motor system after stroke. Previous studies have found that stroke patients showed decreased nodal efficiency, betweenness centrality and degree in the SMA [22]. The functional role of the SMA in healthy subjects has been investigated. This region may participate in motor control and execution and is considered as a predominant hub [43, 44]. For patients with stroke, the most common symptom is motor deficit. Previous neuroimaging studies have found significant disturbances in the effective and functional connectivity of motor areas in stroke patients [18, 45–47]. The intrinsic neural coupling between SMA and M1, and the coupling between the bilateral SMA, was significantly reduced after stroke. A recent study used brain entropy to evaluate motor impairment after stroke and found that stroke patients exhibited a decrease of brain entropy in the bilateral dorsolateral frontal gyrus and SMA [48]. An analysis on electroencephalographic data found that stroke patients exhibited decreased neural activity in the SMA at rest [49]. The functional neural basis in the SMA was altered after stroke. The consistent results with regard to the SMA between the present and these previous studies confirm the important role of SMA in the reorganization of the motor-related regions in stroke patients. As for middle temporal gyrus, this region is associated with the perception of movement within the visual field. Lesions of this region can induce movement agnosia. Previous studies have also found the right superior and middle temporal gyrus to be associated with anxiety at one month after stroke [50]. A structural MRI study found that stroke patients showed significant changes in cortical thickness in the frontal and temporal cortices within two weeks after stroke [51]. Significant decreases in Cp and nodal local efficiency in the right MTG may reflect behavioral functional deficits after stroke.

Other regions, such as the left putamen, left caudate and right temporal pole of the STG, showed significant reductions in nodal efficiency in this study. Nodal efficiency in the left PCG showed significant increase after stroke. Extensive changes in nodal efficiency in the brain indicate that changes in nodal topology present beyond the motor-related network. The left PCG is part of the DMN. Changed brain connectivity in the DMN has been confirmed in stroke patients [41]. Previous studies have also found that the functional connectivity between the frontoparietal network and the DMN showed a significant increase in stroke patients [9]. The left PCG was recruited to compensate for the disruption of connectivity efficiency in the left caudate, left putamen and right temporal pole of the STG after stroke. Thus, the nodal efficiency was enhanced in the left PCG. Although we provide a simple explanation of the nodal efficiency results with simultaneous decreases and increases, further study should be conducted in the future.

### The process of recovery of topological organization after stroke

The aim of this study is to assess longitudinal changes in the topological organization of the brain structural network in stroke. As stroke patients demonstrated behavioral recovery within a one-month interval, the decreased Cp and nodal local efficiency in the SMA and temporal pole also improved one month later and showed no significant differences compared with the HCs. The values of nodal efficiency in the right postcentral gyrus and right IPL were also enhanced one month later in stroke patients. Previous studies have found a beneficial role of motor-related areas on the recovery of motor function following stroke [7, 51, 52]. Additionally, the connectivity related to these motor-related areas was improved and tended toward connectivity values of healthy participants after clinical intervention [11, 53, 54]. Regarding the bilateral SMA, previous studies have shown that these regions are mainly involved in motor planning and motor execution during voluntary movement [55]. The decreased nodal properties in the bilateral SMA in the present study supported this view. One month later, the disrupted nodal properties showed recovery to the level of no significant difference compared with those of the HCs. The significant changes in nodal properties in these regions from the first to the second timepoint can be explained based on the recovery mechanism. During the recovery of patients’ behavioral function, the previous excitatory links connecting these regions were enhanced or new links connecting these regions were created [12]. As a result, the Cp, nodal local efficiency and nodal efficiency showed an increase in these regions.

In this study, we also found that the increased nodal efficiency in the left PCG of stroke patients was reduced to nearly normal levels at the second timepoint compared with that of the HCs. As the PCG belongs to the DMN, stronger nodal efficiency in this region may compensate for the damage to other regions after stroke [41, 56]. At the second timepoint, behavioral function in the patients showed recovery. During this process, the disrupted nodal properties in the bilateral SMA, right temporal pole of the MTG, right postcentral gyrus and right IPL were increased. This reorganization of the cortical functional network may result in a decrease in recruitment of the PCG. Future studies should further investigate this possibility.

Although the findings of this study demonstrated that the brain structural network in chronic stroke patients showed plasticity, the specific mechanism of brain plasticity in this study is still unidentified. Because the present study only included one stroke group with antiplatelet therapy, patients without treatment or with placebo treatment were not included in this study. The present design cannot determine whether graphical changes after intervention were the result of antiplatelet therapy only. Spontaneous recovery can be seen in the first few months after stroke, enabling brain plasticity [3, 57]. The design of the present study cannot exclude spontaneous recovery. Future research should consider this question.

### Clinical relevance of network alterations in adults with stroke

A growing body of evidence has shown that brain changes after stroke may predict the recovery of behavioral function [8, 21, 46, 58]. A previous study in stroke demonstrated that the resting state small-world properties in EEG gamma band correlates with recovery, which can be considered as a biomarker of functional recovery [59]. In this study, we used the graph theory on the structural image to detect whether cortical structural connectivity can reflect stroke patient’s recovery. Significant behavioral-neuroimaging correlations were found in right SMA and right IPL. Only detected in these regions might relate to their specific function in the brain. Liu et al. [60] reported that the integrity of fibers connecting SMA showed the most significant correlation with motor deficits, and is thus useful for assessing and predicting long-term motor outcome in patients with subcortical stroke. A previous study using voxel-based morphometry on structural MRI found that gray matter volume in the contralesional SMA was positively correlated with the motor function in patients after local subcortical infarction [5]. Graphical analysis based on a functional network found that the network connections for areas related to frontoparietal control systems and sensorimotor functions in stroke patients were broken down [27]. Functional network complexity was correlated with hand and wrist function in stroke patients. Topological network changes in stroke patients can reflect the severity of disruption to the structural network and are associated with patients’ behavioral function [21]. Similar findings have been reported by previous studies showing that the SMA is involved in recovery from stroke and that the initial activation in the SMA has some correlations with poststroke motor recovery [61, 62]. A recent study by Liang et al. [48] used brain entropy to explore alterations in complexity after stroke and found that patients showed decreased brain entropy in the SMA. The brain entropy values in the ipsilesional SMA were positively correlated with FMA scores. Consistent with these previous studies, our significant correlation results between nodal properties (Cp and nodal local efficiency) in the right SMA and clinical scores (FMA and NDS) suggest that SMA characteristics are crucial for assessing motor rehabilitation. The present study also found that nodal efficiency in the right IPL was significantly correlated with clinical scores of FMA and NDS in stroke patients. This result is consistent with the results from previous studies. A previous graph theory study of structural networks found dramatically positive correlations between nodal degree of the IPL and FMA scores [22]. The significant relationships between the clinical scores and the nodal properties in the right SMA and IPL may provide potential biomarkers for predicting recovery after stroke.

The novelty of the present study is that longitudinal changes of structural brain connectomes were detected in adults with ischemic stroke. Altered topological properties were found in stroke patients at nodal levels, which can be modulated during recovery. The longitudinal results may provide information for understanding brain recovery from stroke. Another novelty of the present study is the significant relationship between the clinical scores and the nodal properties in some regions (right SMA and right IPL). The results in this study provide preliminary evidence that the disrupted topological organization of the structural network in stroke patients may improve during the recovery process. The nodal properties in these regions may represent a biomarker of recovery in stroke.

### Limitations

There are several issues that need to be addressed. First, all patients received antiplatelet therapy. This study lacked a control patient group who received placebo treatment or did not receive treatment. To identify and verify the effect of longitudinal changes on brain structural network properties purely due to antiplatelet therapy, the design of this study should be optimized in the future. Second, lesion locations and lesion sizes in our sample of patients were not homogeneous. The range of duration from stroke onset to first MRI is broad. All these factors may add variability to the structural connectivity measures. Future studies should recruit larger numbers of patients and divide the patients into different subgroups based on lesion locations. Third, the registration results in the preprocessing were checked by manually viewing the images. Objective methods should be introduced to judge the registration results in the future. Finally, the sample size was relatively small in the present study. Future studies should recruit larger numbers of patients to perform further investigations.

## Conclusion

In this study, we used graph theoretical analyses to investigate stroke-related changes in the topological organization of structural network in adults with subcortical stroke. Although the structural connectivity networks in stroke patients still maintained small-world topology, as in HCs, the structural network in stroke patients also exhibited significant changes in nodal properties. Significant decreases in both nodal local efficiency and Cp of patients were found in the right SMA, right MTG and right temporal pole of the MTG. Stroke patients also exhibited decreased nodal efficiency in the left putamen, left caudate and right temporal pole of the STG and increased nodal efficiency in the left PCG. Longitudinal comparison indicated that these network changes in the regions mentioned above recovered to a level not significantly different from that of the HCs. In addition, nodal graph properties in the right SMA and right IPL were related to the clinical scores of FMA and NDS. The correlation results suggest that the values of nodal graph properties in the right SMA and IPL may reflect the patient’s motor function and the degree of neurological functional deficit. Longitudinal changes in topological properties in structural networks may provide information useful for understanding brain recovery from stroke. Future studies should determine whether brain connectome altered as observed in this study can predict the recovery of day-to-day cognitive function following stroke.

## Supplementary Information

**Additional file 1: Figure S1.** The changes of clinical variables in stroke patients with intervention. FMA, Fugl-Meyer motor assessment; NDS, neurological deficit scores.

## Data Availability

The data that support the findings of this study are available from the corresponding author upon reasonable request.
