# Supplementary figures and images for: The disrupted topological properties of structural networks showed recovery in ischemic stroke patients: a longitudinal design study
Source: BMC Neurosci. 2021 Aug 2;22:47. doi: 10.1186/s12868-021-00652-1 (PMC8330082; doi:10.1186/s12868-021-00652-1)

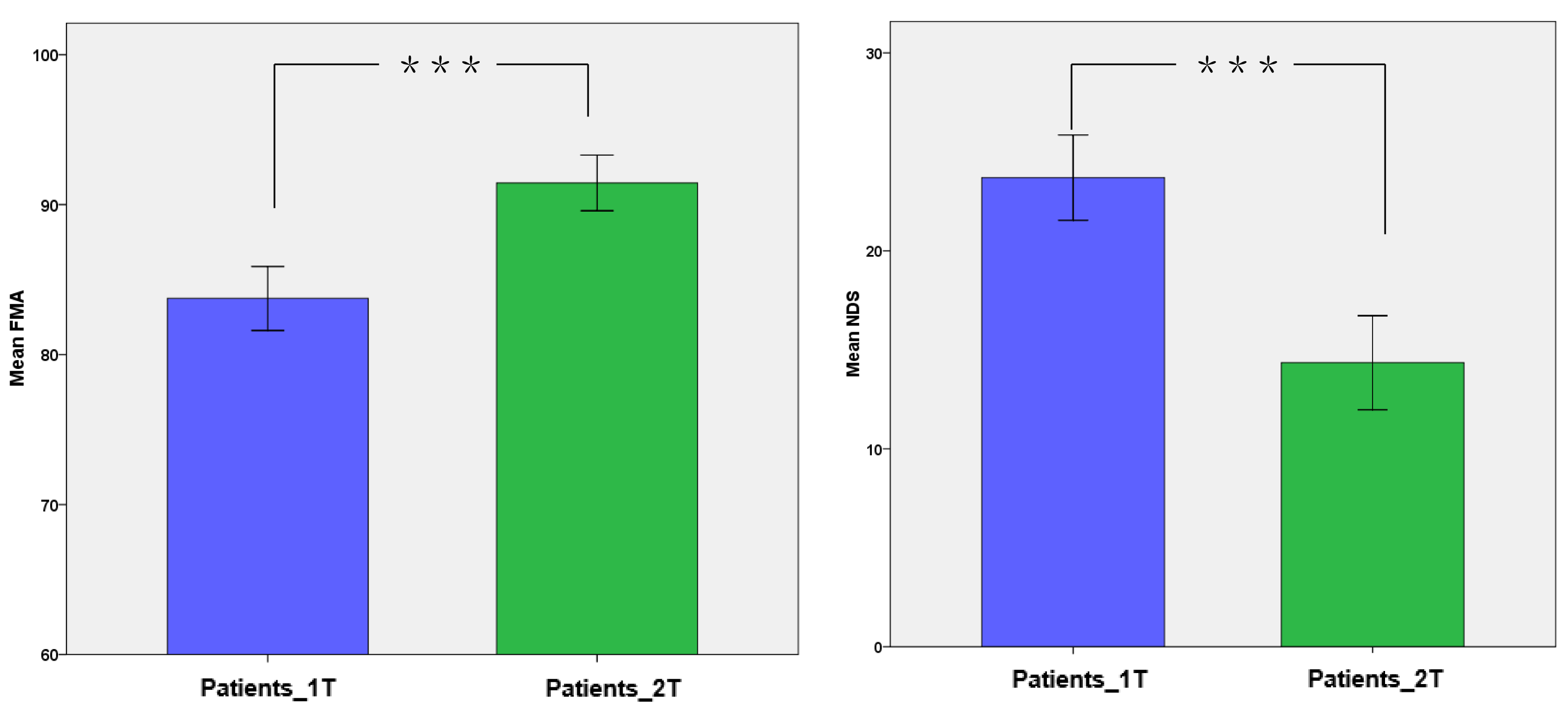

Supplement: Supplementary file 1 — Additional file 1: Figure S1. The changes of clinical variables in stroke patients with intervention. FMA, Fugl-Meyer motor assessment; NDS, neurological deficit scores. [file 12868_2021_652_MOESM1_ESM.tif]
